# High Constitutive Overexpression of Glycosyl Hydrolase Family 17 Delays Floral Transition by Enhancing FLC Expression in Transgenic *Arabidopsis*

**DOI:** 10.3390/plants6030031

**Published:** 2017-07-25

**Authors:** Shinichi Enoki, Nozomi Fujimori, Chiho Yamaguchi, Tomoki Hattori, Shunji Suzuki

**Affiliations:** Laboratory of Fruit Genetic Engineering, The Institute of Enology and Viticulture, University of Yamanashi, Yamanashi 400-0005, Japan; senoki@yamanashi.ac.jp (S.E.); noro.noro.n@live.jp (N.F.); g16lf017@yamanashi.ac.jp (C.Y.); g15de002@yamanashi.ac.jp (T.H.)

**Keywords:** grapevine, β-1,3-glucanase, inflorescence, *VvGHF17*, floral transition, *FLC*, *Arabidopsis*

## Abstract

*Vitis vinifera* glycosyl hydrolase family 17 (VvGHF17) is a grape apoplasmic β-1,3-glucanase, which belongs to glycosyl hydrolase family 17 in grapevines. β-1,3-glucanase is not only involved in plant defense response but also has various physiological functions in plants. Although *VvGHF17* expression is negatively related to the length of inflorescence in grapevines, the physiological functions of *VvGHF17* are still uncertain. To clarify the physiological functions of *VvGHF17*, we conducted a phenotypic analysis of VvGHF17-overexpressing *Arabidopsis* plants. VvGHF17-overexpressing *Arabidopsis* plants showed short inflorescence, similar to grapevines. These results suggested that *VvGHF17* might negatively regulate the length of inflorescence in plants. *VvGHF17* expression induced a delay of floral transition in *Arabidopsis* plants. The expression level of *FLOWERING LOCUS C* (*FLC*), known as a floral repressor gene, in inflorescence meristem of transgenic plants were increased by approximately 10-fold as compared with wild plants. These results suggest that *VvGHF17* induces a delay of floral transition by enhancing *FLC* expression and concomitantly decreases the length of plant inflorescence.

## 1. Introduction

Higher plants such as vascular plants have certain defense mechanisms against plant pathogens such as fungi, bacteria, and viruses. Pathogenesis-related proteins (PRs) are induced against pathogen invasion in plants and play an important role in plant defense [1]. PRs are classified into 17 families (PR-1 to PR-17) according their characteristics [2]. β-1,3-Glucanases (glucan endo-1,3-β-d-glucosidase, EC 3.2.1.39) belong to the second (PR-2) of the 17 families and are also included in the glycosyl hydrolase family 17 (GHF17) due to their degradation style. Since they hydrolyze 1,3-β-d-glycosidic bonds in β-1,3-glucan as the main component of the cell wall of many fungi [3,4], β-1,3-glucanases are thought to play an important role in the plant defense response against pathogen infection. In our previous study [5], we isolated the apoplasmic β-1,3-glucanase secreted from grape cells and demonstrated that VvGHF17-overexpressing *Arabidopsis thaliana* acquired multiple resistance to phytopathogenic fungi.

β-1,3-glucan (referred to as callose in plants) is widespread in plant bodies. Thereby, β-1,3-glucanase has various physiological functions in addition to plant defense, such as cell division and elongation [6,7], flower formation [8,9], pollen germination and tube growth [10], fertilization [11], and fruit ripening [12,13]. In particular, understanding the influence of β-1,3-glucanase on traits such as flower formation and subsequent fruit ripening is very important, since their traits are directly linked to the fruit quality which determines the value of fruit trees. However, there are few reports that β-1,3-glucanase has physiological functions affecting plant growth in grapevines, and its physiological functions are still uncertain.

To understand the physiological functions of *VvGHF17* in the vegetative and/or reproductive growth of grapevines, we conducted a phenotypic analysis of VvGHF17-overexpressing *Arabidopsis* plants. The present study demonstrates that *VvGHF17* delays floral transition in *Arabidopsis* plant through enhancing *FLOWERING LOCUS C* (*FLC*) expression, which is the transcription factor functioning as a repressor of floral transition.

## 2. Results

### 2.1. VvGHF17 Expression Is Negatively Related to the Length of Grape Inflorescence

**Figure 1:**
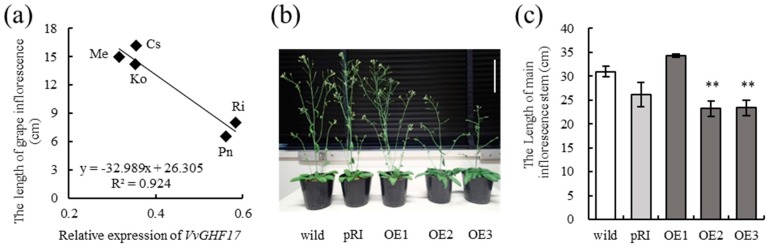
*VvGHF17* expression suppresses inflorescence growth in plants. (**a**) Regression line between the length of mature grape inflorescence and endogenous *VvGHF17* expression in young grape inflorescence. Averages (*n* = 10) are plotted in the graph. Cs, Cabernet Sauvignon; Ko, Koshu; Me, Merlot; Pn, Pinot Noir; Ri, Riesling grapevine cultivars. (**b**) VvGHF17-overexpressing *Arabidopsis* plants. Photograph was obtained at 31 days after sowing. Scale bar = 7.5 cm. (**c**) Length of main inflorescence of VvGHF17-overexpressing *Arabidopsis* plants. Bars indicate means ± standard errors (*n* = 5). ** *p* < 0.01 as compared with wild plants.

A simple linear regression analysis was performed to investigate the relationship between the inflorescence length and the gene expression level of endogenous *VvGHF17* in grape cultivars. Endogenous *VvGHF17* expression in young grape inflorescence showed a strong negative correlation with the length of mature grape inflorescence (*p* = 0.0091) (Figure 1a). This result suggested that *VvGHF17* might function in the vegetative and/or reproductive growth of grapevines.

### 2.2. VvGHF17 Induce Delays of Floral Transition

**Figure 2:**
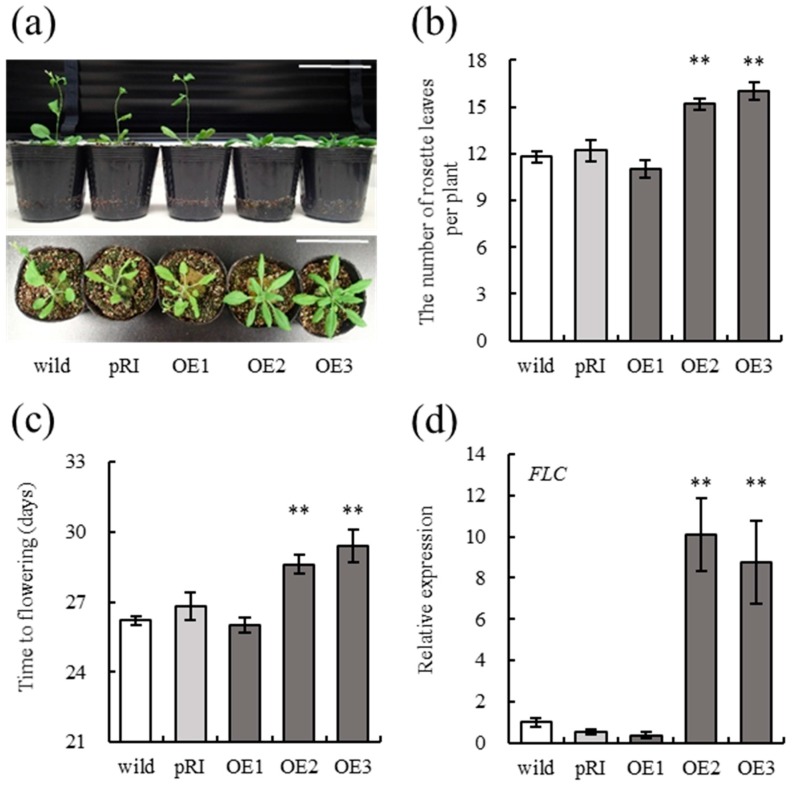
*VvGHF17* delays floral transition by enhancing *FLC* expression in VvGHF17-overexpressing *Arabidopsis* plants. (**a**) Photograph of VvGHF17-overexpressing *Arabidopsis* plants at 26 days after sowing. Scale bar = 7.5 cm. (**b**) Number of rosette leaves formed before the appearance of the inflorescence meristem. (**c**) Time to flowering. Bars indicate means ± standard errors (*n* = 5). (**d**) *FLC* expression. Total RNA was isolated from inflorescence meristems of 20-day-old *Arabidopsis* plants and subjected to real-time RT-PCR analysis. Bars indicate means ± standard errors (*n* = 12). ** *p* < 0.01 as compared with wild plants.

The phenotypic analysis of VvGHF17-overexpressing transgenic *Arabidopsis* obtained by our previous study [5] was performed to clarify the detailed physiological function of *VvGHF17*. The growth of the main inflorescence stem of VvGHF17-overexpressing *Arabidopsis* plants (OE2 and OE3) tended to be poor (Figure 1b). The length of these stems was significantly lower than those of wild plants at 31 days after sowing (Figure 1c). In addition, the number of rosette leaves of transgenic plants (OE2 and OE3) was significantly higher compared with wild plants (Figure 2a,b). *VvGHF17* induced a delay of floral transition in VvGHF17-overexpressing *Arabidopsis* plants (Figure 2c). These results indicate a delay of floral transition in VvGHF17-overexpressing *Arabidopsis* plants and a lower the elongation of their inflorescence.

### 2.3. VvGHF17 Upregulates FLOWERING LOCUS C Expression

To determine the molecular mechanism on the delay of floral transition in the VvGHF17-overexpressing *Arabidopsis* plants, we analyzed the expression level of the *FLC* gene, which is a floral repressor gene [14], in each plant. *FLC* expression levels in OE2 and OE3 were increased by 10.12- and 8.76-fold compared to those of wild plants, respectively (Figure 2d). This result suggests that *VvGHF17* delays floral transition through the alternation of floral repression.

## 3. Discussion

We demonstrated that *VvGHF17* induces floral transition in *Arabidopsis* plants. The number of rosette leaves formed before the appearance of the inflorescence meristem was measured as an indicator of floral transition, because there is a positive correlation between the number of rosette leaves and floral transition [15]. The number of rosette leaves in VvGHF17-overxpressing plants (OE2 and OE3) was higher compared with that of wild plants (Figure 2a,b). Flowering day was delayed as well (Figure 2c). These results indicate that the vegetative growth period of VvGHF17-overexpressing *Arabidopsis* plants became longer than those of wild plants. However, there was no significant difference between OE1 and wild plants in this study. This may be due to the fact that the expression level of *VvGHF17* in OE1 is much lower than those of OE2 and OE3 in our previous report [5].

**Figure 3:**
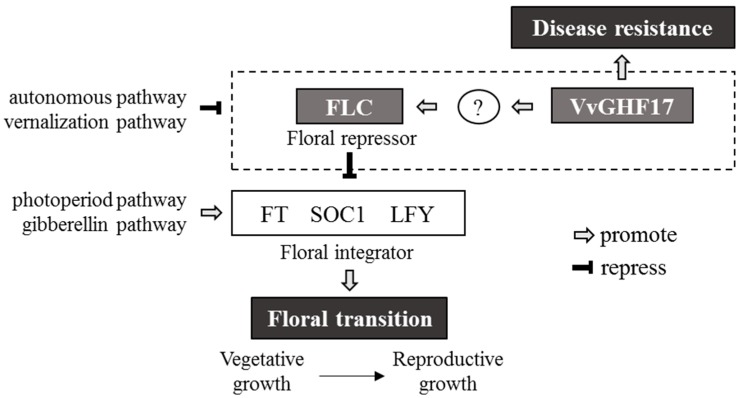
Theoretical model of VvGHF17-mediated delay of floral transition. Floral transition is controlled through four pathways: autonomous, vernalization, photoperiod, and gibberellin pathways. *FLC* encodes a MADS domain protein and integrates signal through autonomous and vernalization pathways. FLC acts as a repressor of flowering. VvGHF17 enhances the expression level of FLC directly or indirectly by an unknown mechanism. FLC represses the expression levels of SOC1, FT, and LFY, which are floral integrators. Therefore, VvGHF17 delays floral transition. FLC, FLOWERING LOCUS C; SOC1, SUPPRESSOR OF CO OVEREXPRESSION 1; FT, FLOWERING LOCUS T; LFY, LEAFY.

Figure 3 shows the relationship between the VvGHF17 and floral transition suggested from this study. Floral transition is controlled through four pathways: autonomous, vernalization, photoperiod, and gibberellin pathways. *FLC* encodes a MADS domain protein, which conserved sequence motif with many transcription factors, and integrates signals through autonomous and vernalization pathways. FLC acts as a repressor of flowering [15]. VvGHF17 enhances the expression level of FLC directly or indirectly by an unknown mechanism. SUPPRESSOR OF CO OVEREXPRESSION 1 (SOC1), FLOWERING LOCUS T (FT), and LEAFY (LFY) are known as floral integrators and are upregulated by photoperiod and gibberellin pathways, respectively [16,17]. The expression of FLC downregulates the expression levels of SOC1, FT, and LFY. Therefore, FLC upregulation in VvGHF17-overexpressing plants induces a delay of floral transition. This is the first report, to our knowledge, that β-1,3-glucanase affects the timing of floral transition. However, since FLC is controlled by many genes in the autonomous and vernalization pathways [18], it is still unclear, according to this study, whether VvGHF17 directly or indirectly controls FLC through any of the genes of these two pathways.

Endogenous GHF17 is highly expressed at the stage of flower formation [8] and fruit ripening [12]. Thus, multifunction of β-1,3-glucanase in vegetative and/or reproductive growth and in floral transition remains still unclear. So far, we could not demonstrate any mechanisms from *VvGHF17* expression to *FLC* expression. On the other hand, β-1,3-glucanase hydrolyses β-1,3-glucans is not only from fungal cell walls, but also from callose in plants. Flax overproducing β-1,3-glucanase changes cell wall composition and shows a decrease in callose (endogenic β-1,3-glucan) content as well as an increase in particular polysaccharides contents [19]. Therefore, the delay of floral transition in VvGHF17-overexpressing *Arabidopsis* plants might be due to the change of plant cell wall composition by *VvGHF17*, resulting in a change in cell growth. Further studies employing transcriptional analyses of genes located upstream of *FLC* in addition to surveys of cell wall composition in inflorescence would reveal the molecular mechanisms of the relationship between *VvGHF17* and floral transition.

*VvGHF17* has the function of multiple disease resistance against phytopathogenic fungi [5]. Generally, the longer the period of vegetative growth in grapevines, the higher the quality of the grape berries. Therefore, the production of VvGHF17-overexpressing grapevines could lead to the breeding of grapevines with disease resistance and good fruit quality. Although we revealed that *VvGHF17* influences floral transition, the influence of *VvGHF17* on fruit traits after flowering such as yield and fruit quality are still unknown. In the future, field research focusing disease resistance and quality and quantity of grape berries on VvGHF17-overexpressing grapevines would be required for an evaluation of genetically engineered grapevines.

## 4. Materials and Methods

### 4.1. Plant Materials

Grape cultivars in the test field of The Institute of Enology and Viticulture, University of Yamanashi (Japan), were used as plant materials. Grape bunches of each grapevine cultivar (*Vitis vinifera* cvs. Cabernet Sauvignon, Koshu, Merlot, Pinot Noir, and Riesling) were collected at young and mature stages in 2015. The length of mature grape inflorescences was measured.

*Arabidopsis thaliana* wild type (Col-0), pRI101-AN vector-transformed *Arabidopsis* plants (pRI) and VvGHF17-overexpressing *Arabidopsis* plants (OE1, OE2, and OE3), which were obtained in our previous study [5], were used as plant materials. T3 (third generation transgenic plant) homozygote seeds were sown in rockwool (2.5 cm × 2.5 cm × 3.8 cm) and grown in an incubator (11.8 W^−2^/16 h/day, 22 °C). One week after sowing, the seedlings were moved to the soil together with the rock wool and grown in the incubator under same conditions.

### 4.2. Phenotypic Analysis in Arabidopsis Plants

After sowing each *Arabidopsis* plant, their phenotypes were observed daily. The length of the main inflorescence stem at 4 weeks after sowing, as well as the number of rosette leaves per plant at the appearance of an inflorescence, and the time to flowering was measured.

### 4.3. Isolation of Total RNA

We isolated the total RNA from the inflorescence meristem of grape and *Arabidopsis* plants. Young grape inflorescences of 3–8 mm in the longitudinal direction of each cultivar were used. The inflorescence meristems of 20-day-old *Arabidopsis* plants before the appearance of an inflorescence were used. After freezing these samples with liquid nitrogen, the samples were homogenized with an SK mill (SK-200) (Tokken, Kashiwa, Japan). According to the manufacturer’s instructions, total RNA was isolated from these homogenized samples using Nucleospin RNA plant (Takara, Otsu, Japan).

### 4.4. Real-Time RT-PCR

First-strand cDNA were synthesized from the total RNA using a PrimeScript RT Reagent Kit with gDNA Eraser (Takara) and subsequently used for real-time (RT)-PCR analysis. RT-PCR analysis was performed using an SYBR Premix Ex Taq II (Takara) by Thermal Cycler Dice Real-Time System Single Software ver. 3.00 (Takara) and the standard curve method. Reaction conditions were as follows: 37 °C for 15 min, 85 °C for 5 s, 40 cycles at 95 °C for 5 s, and 60 °C for 30 s. Primer sequences were as follows: *Arabidopsis* FLC primers (5′-GAGCCAAGAAGACCGAACTCA-3′ and 5′-TCTCAGCTTCTGCTCCCACA-3′, GenBank accession no. NM_121052) and *Arabidopsis* actin primers (5′-GCCGACAGAATGAGCAAAGAG-3′ and 5′-AGGTACTGAGGGAGGCCAAGA-3′, GenBank accession no. NM_179953). *VvGHF17* primers were used as described previously [5]. *FLC* and *VvGHF17* expression levels were normalized to each *actin*, and relative expression of *FLC* in *Arabidopsis* plants were represented as values relative to the controls (wild plants).

### 4.5. Simple Linear Regression Analysis

Simple linear regression analysis between the length of mature grape inflorescence and the relative expression of *VvGHF17* in young grape inflorescence was conducted using Excel statistics software 2012 (Social Survey Research Information, Tokyo, Japan). The dependent and explanatory variables were the length of grape inflorescence and the relative expression of *VvGHF17*, respectively.

### 4.6. Statistical Analysis

The data are shown as means ± standard errors in the tests with *Arabidopsis* plants. These data were statistically analyzed by Dunnett’s multiple comparison test using Excel statistics software 2012 (Social Survey Research Information, Tokyo, Japan).
